# Integrating Machine Learning and Expert Sensory Evaluation to Identify Key Drivers of Tomato Fruit Quality: A Multi-Model and Age-Stratified Analysis

**DOI:** 10.3390/foods15132358

**Published:** 2026-07-02

**Authors:** Yihang Zhu, Chenxu Liu, Zhuping Yao, Rongqing Wang, Baoliang Xie, Yuan Cheng, Xiaobin Zhang

**Affiliations:** State Key Laboratory for Quality and Safety of Agro-Products, Institute of Digital Agriculture, Vegetable Research Institute, Zhejiang Academy of Agricultural Sciences, Hangzhou 310021, China; zhuyh@zaas.ac.cn (Y.Z.); liuchenxu@zaas.ac.cn (C.L.); yaozp@zaas.ac.cn (Z.Y.); wangrq@zaas.ac.cn (R.W.); m15858327936@163.com (B.X.)

**Keywords:** tomato fruit quality, random forest, age stratification, flavor-related metabolites, precision breeding

## Abstract

Individual biochemical indicators are insufficient for comprehensive tomato food flavor quality assessment, necessitating multi-parameter models of the core soluble taste matrix. We hypothesized that age stratification of trained sensory assessors would expose differential biochemical variable importance profiles in flavor quality prediction. Accordingly, this study aimed to: (1) construct and compare multiple regression models linking eight biochemical indicators to sensory scores, (2) identify key quality drivers via feature selection, and (3) examine whether age stratification alters the identified sensory drivers. Eight baseline taste indicators across 62 tomato cultivars were evaluated by 30 age-stratified trained sensory panelists (<40 and ≥40 years), using cross-validation to ensure model robustness against small-sample constraints. Partial least squares regression (PLSR), support vector regression (SVR), random forest (RF), and Boruta were applied. Random forest achieved the best performance (R^2^ = 0.82). In the full panel model, key variables were fructose, total free amino acids, and vitamin C. After age stratification, the under-40 group retained these variables, whereas the ≥40 group replaced vitamin C with soluble solids. Fructose and total free amino acids were consistently robust drivers, while total acidity remained least important. Deploying the RF–Boruta framework within an age-stratified context provides a structured analytical framework for investigating flavor perception from biochemical data. These findings suggest that fructose and total free amino acids represent highly robust candidate indicators for flavor quality prediction, while age-stratified variances suggest the utility of integrating demographic-specific metrics into precision breeding frameworks.

## 1. Introduction

Tomato (*Solanum* *lycopersicum* L.) is one of the most widely cultivated and economically significant vegetable crops globally. As an important dietary source of nutrients, a tomato is rich in vitamins, carotenoids, and a range of antioxidant compounds. Notably, its sensory quality, particularly flavor, directly determines consumer purchasing intent and market competitiveness. However, over the past few decades, modern commercial breeding programs have prioritized yield, fruit firmness, and postharvest storability, resulting in a substantial deterioration of tomato flavor quality [1]. Consumers have widely reported a loss of ‘tomato-like’ taste, which has become a key bottleneck constraining industry upgrading and failing to meet escalating demands for high-quality produce [2]. Therefore, systematically improving tomato flavor quality while maintaining yield and stress resistance has emerged as a critical research priority in both plant breeding and food science [3]. Current research has turned to comprehensive metabolomics and volatile organic compound (VOC) profiling to capture the multi-dimensional networks that define flavor perception [4]. Concurrently, as the food sector increasingly adopts complex predictive architectures, the deployment of explainable artificial intelligence (XAI) frameworks, such as feature attribution methods, SHAP values, has become critical [5]. These approaches transition agricultural predictive modeling from opaque ‘black boxes’ into transparent, interpretable systems capable of extracting actionable biological insights for targeted breeding.

The formation of tomato flavor quality is a complex physiological and biochemical process involving the coordinated action of multiple metabolites, including soluble sugars, organic acids, amino acids, and volatile aroma compounds [6]. Optimal flavor perception depends on a balanced multi-dimensional sensory experience encompassing sweetness, acidity, and umami. While volatile organic compounds (VOCs) are vital for the olfactory component of tomato flavor, the non-volatile soluble matrix, including a core group of low-molecular-weight metabolites, comprises fructose, glucose, organic acids, vitamin C, and free amino acids, and establishes the baseline taste perception (sweetness, acidity, and umami) upon which aromatic notes interact. Consequently, evaluating these primary taste indicators provides a foundational mapping of sensory-driven palatability [2]. In current breeding and production practices, tomato quality assessment still heavily relies on single indicators, most commonly soluble solids content (°Brix), or at best employs the sugar–acid ratio as a simplified proxy. Although operationally convenient, this approach evidently fails to capture the full complexity of flavor. The sensory panel, widely regarded as the ‘gold standard’ for flavor evaluation, can systematically integrate multiple sensory dimensions and provide assessments most closely aligned with real consumer perception [7]. Nevertheless, this method also has notable limitations: high subjectivity, substantial organizational costs, time intensity, and susceptibility to panelist composition [8]. Of particular relevance, significant age-related differences in taste sensitivity have been documented across its lifespan, with the fourth decade (approximately 40 years of age) serving as a distinct physiological inflection point. At this developmental threshold, the homeostatic regeneration rate of taste receptor cells within the fungiform and circumvallate papillae begins a progressive decline, leading to elevated absolute detection thresholds and altered suprathreshold intensity perception for primary modalities such as sweetness and acidity [9,10]. Consequently, if breeding programs target diverse consumer groups yet rely on sensory evaluation systems that do not account for age structure, the resulting guidance may be subject to systematic bias.

In recent years, the rapid advancement of machine learning has provided new methodological tools for predictive modeling of complex traits. Compared with conventional statistical methods, machine learning models can effectively capture nonlinear relationships and interactive effects within high-dimensional datasets, demonstrating considerable potential in fruit quality prediction. Existing studies have explored regression models for predicting tomato flavor-related attributes; however, most investigations have focused on single-model applications or simple model comparisons [11]. While machine learning approaches have been successfully deployed to predict the sensory quality and consumer acceptance of other major fruit crops like strawberries, peaches, and apples using metabolic or spectral profiles, systematic performance multi-model comparisons across diverse architectures, such as linear models (e.g., partial least squares regression, PLSR), kernel-based methods (e.g., support vector regression, SVR), and ensemble learning approaches (e.g., random forest, RF), remain limited in tomato sensory modeling [12,13]. Furthermore, in feature selection, conventional studies predominantly employ simple correlation-based methods to identify key quality indicators, which are vulnerable to multicollinearity interference and struggle to isolate factors with genuinely independent contributions to flavor perception [7]. While ensemble learning and wrapper-based feature selection methods like the Boruta algorithm have been utilized independently in agricultural modeling, their deployment within an age-stratified sensory framework exposes distinct, age-dependent metabolic pathways that govern taste perception across different consumer generations [14].

More critically, although prior research has acknowledged the influence of age on taste perception, studies applying age-stratified sensory evaluation and quality indicator association analysis in fruit quality modeling remain largely absent [9,10]. For breeders, if the target market is predominantly younger or middle-aged consumers, relying on evaluation models calibrated on all-age or age-unstructured panels may result in a mismatch between breeding targets and the sensory expectations of the intended demographic [15]. Therefore, incorporating age stratification into tomato flavor quality prediction models and systematically comparing the responses of different age groups to identical quality indicators not only addresses a theoretical gap but also holds substantial practical value for market-segmented breeding programs [11,16].

Against this backdrop, this study was designed to address three specific objectives: (1) construct and compare multiple regression models linking eight primary non-volatile biochemical indicators to trained panel sensory scores of tomato flavor quality; (2) identify the key biochemical drivers of sensory quality and evaluate their consistency among different age groups; and (3) examine whether age stratification of the sensory panel (<40 vs. ≥40 years) leads to structurally distinct variable importance profiles, which would indicate that the relative contribution of biochemical indicators to perceived flavor quality is age-dependent. Accordingly, we hypothesize that the age-stratified subgroup models would exhibit differential Boruta variable importance rankings relative to the full-panel model, with the <40 and ≥40 year subgroups showing at least one non-overlapping confirmed variable. To test these objectives, 62 tomato germplasm accessions were evaluated by 30 trained sensory assessors stratified by age, and eight baseline taste-related biochemical indicators were quantified.

## 2. Materials and Methods

### 2.1. Plant Materials

A total of 62 tomato germplasm accessions were collected in this study, encompassing cultivated varieties, landraces, and several wild relatives. These accessions were specifically selected based on documented variations in fruit morphology, pigmentation, and baseline quality profiles, detailed in Supplementary Table S1. All plant materials were cultivated in the spring of 2023 at the experimental station of Zhejiang Academy of Agricultural Sciences (30.3° N, 120.2° E), under uniform irrigation, fertilization, and pest management protocols. Ten plants per accession were grown in a randomized complete block design with three replicates, using a plant spacing of 40 cm × 70 cm. Fruits from the second and third trusses were harvested at the ripening stage according to the GH/T 1193-2021 [17] tomato standard (color transition completely to red, fruit became soft and elastic) before noon to eliminate diurnal physiological fluctuations, selecting only undamaged fruits of uniform size. Harvested fruits were immediately placed in well-ventilated, temperature-insulated containers and transferred within 1 h to a stabilization facility maintained at lower than 15 °C and 75–90% relative humidity to minimize postharvest respiration drifts. Thirty fruits per accession were then collected and immediately processed for sensory evaluation. Tissue aliquots designated for biochemical determination were homogenized, flash-frozen in liquid nitrogen, and kept at −80 °C immediately after sensory evaluation. The whole process was conducted within 4 h.

### 2.2. Sensory Evaluation Design

The trained sensory panel consisted of 30 healthy assessors aged 25–55 years, stratified by age: 14 individuals in the <40 years group (6 males, 8 females) and 16 individuals in the ≥40 years group (7 males, 9 females). This 40-year stratification boundary was selected to align with chemosensory frameworks indicating that middle age marks the onset of gradual, multidimensional sensory drift, thereby effectively isolating peak gustatory performance from the cohort experiencing progressive chemosensory changes. None of the assessors had known taste or olfactory dysfunctions, and they were asked to refrain from consuming pungent foods within 24 h prior to evaluation. Although a total of 30 assessors were utilized, the sensory design was structured as a fully crossed matrix wherein every panelist evaluated all 62 tomato germplasm accessions, and a retrospective power analysis based on the observed cross-group mean profiles was conducted.

Standardized training of panelist instruction was conducted over 2 days (two 2 h sessions per day) prior to evaluation in accordance with the GB/T 29605-2013 standard [18]. Explicit reference standards were utilized during training to calibrate the panel and anchor the 1–5-point scale: aqueous solutions of 5% (*w*/*v*) sucrose-anchored peak sweetness (score 5), 0.1% (*w*/*v*) citric acid-anchored peak acidity (score 5), and 0.2% (*w*/*v*) monosodium glutamate (MSG)-anchored peak umami (score 5), while deionized water served as the baseline (score 0) for all taste modalities. Panel calibration was verified dynamically; panelists were cleared for formal evaluation only when individual scoring variance across blind duplicate reference samples achieved a coefficient of variation (CV) of less than 10%.

All formal evaluations were conducted in a standardized environment: testing took place in isolated individual booths under constant artificial daylight illumination (6500 K), with the ambient room temperature strictly regulated at 23 ± 1 °C and relative humidity maintained at 50–70% to eliminate external environmental bias. A blinded evaluation protocol was adopted, with each sample randomly coded. Deionized water was used as a palate cleanser between samples. Each evaluation round included three replicate samples, and each sample was evaluated three times by each assessor; the arithmetic mean of the three ratings was taken as the final score for that assessor–sample pair. A 1–5-point rating scale with 0.5-point increments was employed, establishing a 9-point distribution profile. This configuration was selected instead of a 9-point hedonic scale or continuous line scales to reduce panelist cognitive fatigue and combat central tendency bias across the large tomato samples (N = 62). By anchoring specific target quality tiers, this interval design stabilizes scoring boundaries across multiple evaluation sessions while maintaining sufficient mathematical variance for machine learning regression. The categorical descriptors were defined as follows: 1, extremely poor flavor, essentially no tomato taste; 2, poor flavor, tomato taste barely perceptible; 3, acceptable flavor, tomato taste acceptable; 4, good flavor, pronounced tomato taste; and 5, excellent flavor, rich and well-balanced tomato taste.

Inter-rater reliability was assessed using Kendall’s coefficient of concordance (W) with a Chi-square asymptotic test *p* < 0.01 level and 95% confidence intervals (CIs) considered significant, for both within-group and between-group agreement. The final sensory score for each sample was computed in two ways: (1) the all-panel mean, calculated as the arithmetic mean across all 30 panelists; and (2) the age-subgroup mean, computed separately for the <40 years and ≥40 years subgroups, and used for age-stratified modeling analyses.

### 2.3. Biochemical Indicator Determination

Five mature fruits were randomly selected from each accession, stemmed and homogenized, and the supernatant was used for biochemical analyses. Eight indicators were quantified, covering sweetness, acidity, umami, and nutritional quality-related factors:

- Soluble solids content (SSC): Measured using a digital refractometer (PAL-1, Atago, Tokyo, Japan), expressed as °Brix, three replicates per sample.
- Soluble sugar (SS): Determined by the anthrone–sulfuric acid colorimetric method, expressed as mg/g fresh weight (FW), three replicates per sample.
- Fructose: Quantified by high-performance liquid chromatography (HPLC; Agilent 1260, Santa Clara, CA, USA), expressed as mg/g FW, three replicates per sample.
- Glucose: Determined using the same HPLC method as for fructose, expressed as mg/g FW, three replicates per sample.
- Total acidity (TA): Measured by acid–base titration (expressed as citric acid equivalent), expressed as %, three replicates per sample.
- Vitamin C (VC): Determined by the 2,6-dichlorophenolindophenol titration method, expressed as mg/g FW, three replicates per sample.
- Total free amino acids (AAs): Quantified by the ninhydrin colorimetric method, expressed as μmol/g FW, three replicates per sample.
- Glutamic acid (GLU): Quantified by HPLC, expressed as mg/g FW, three replicates per sample.

To ensure high analytical rigor and data fidelity for machine learning feature engineering, all biochemical methods were systematically validated for linearity, sensitivity, precision, and accuracy. The limits of detection (LOD) and quantification (LOQ) were determined based on signal-to-noise ratios of 3 and 10, respectively. For HPLC determinations (fructose, glucose, and glutamic acid), LODs ranged from 0.01 to 0.05 mg/g FW, LOQs ranged from 0.03 to 0.15 mg/g FW, relative standard deviation (RSD) was <2.8%, and mean spike recoveries were 94.8–102.1%. For spectrophotometric and titration assays (soluble sugars, total acidity, total free amino acids, and vitamin C), the RSDs were uniformly less than 3.5% and recovery rates spanned 93.5–101.8%, confirming excellent method reproducibility. While the aggregate total free amino acid pool is maintained in molar units (μmol/g FW) to accurately reflect primary amino group stoichiometry, individual glutamic acid is reported as a mass fraction (mg/g FW) in accordance with standard compound-specific chromatographic profiling. Finally, all chemical indicator values were normalized using z-scores prior to model training.

### 2.4. Principal Component Analysis

Principal component analysis (PCA) was conducted as an exploratory step to characterize the overall distribution of the 62 tomato accessions across the eight biochemical indicators. Input data were subjected to z-score standardization. Principal components were extracted based on the covariance matrix, retaining components with eigenvalues greater than 1, and cumulative variance explained was calculated. A biplot was generated to visualize sample distribution and the relationships among biochemical indicators, facilitating the identification of clustering patterns and underlying grouping structures. PCA was performed in R (v4.2.1) using the FactoMineR and factoextra packages.

### 2.5. Model Construction and Evaluation

Three regression models were employed to predict tomato sensory quality ratings based on the eight biochemical indicators: partial least squares regression (PLSR), support vector regression (SVR), and random forest (RF). Model inputs were the eight biochemical indicators; the target variables were the sensory scores (all-panel mean, <40 years subgroup mean, and ≥40 years subgroup mean, respectively).

Given the limited sample size (N = 62), leave-one-out cross-validation (LOOCV) was adopted as the primary model evaluation strategy to avoid overly small training sets. Additionally, 5-fold cross-validation (5-fold CV), repeated three times, was used as a supplementary validation to assess model stability. Although a sample size of 62 is modest for traditional machine learning, ensemble methods like SVM and RF are mathematically robust against overfitting in small-sample contexts due to bootstrap aggregation. To strictly penalize overfitting and ensure generalization, LOOCV was integrated with internal out-of-bag (OOB) error monitoring, which was an unbiased estimate of generalization capacity on unseen data, and entirely excluded from individual tree bootstrap bags. Hyperparameter tuning was performed within the cross-validation framework: the PLSR component number was selected by cross-validation; SVR hyperparameters (kernel type: linear and radial basis function; penalty parameters C and γ) were optimized by grid search; RF hyperparameters (number of trees: ntree = 500, 1000; number of variables per node: mtry = 2, 3, 4) were optimized by grid search. Model performance was evaluated using coefficient of determination (R^2^), root mean squared error (RMSE), and mean absolute error (MAE). For the random forest model, the OOB error was also reported as an unbiased estimate of generalization performance, serving as a safeguard against overfitting in the small-sample context. While these strict internal resampling workflows (LOOCV and repeated 5-fold CV) maximize data efficiency and offer conservative error bounds in the absence of an independent external validation dataset, they primarily demonstrate performance boundaries within the current germplasm pool.

### 2.6. Age-Stratified Modeling

To investigate the effect of assessor age on sensory evaluation models, separate PLSR, SVR, and RF models were independently constructed using the <40 years subgroup mean scores and the ≥40 years subgroup mean scores as target variables, respectively. The same 62 accessions and the same eight biochemical indicators were used as inputs. Model construction and evaluation procedures were identical to those described in Section 2.5. By comparing the prediction performance (R^2^, RMSE, MAE) between the two age-group models, the extent to which age stratification modulates quality prediction was assessed.

### 2.7. Boruta Feature Selection

To identify key biochemical drivers of tomato flavor quality and to compare their importance across age subgroups, the Boruta algorithm was applied for feature selection. Boruta is a wrapper algorithm built upon random forest that constructs shadow features (permuted copies of all original features) and iteratively compares the importance of original variables against shadow features using z-score-based significance testing. Variables whose importance significantly exceeds that of shadow features are classified as ‘confirmed.’

Boruta analysis was run separately for each age subgroup, using the corresponding sensory scores as the target variable. Parameter settings were: number of iterations = 1000, and significance threshold *p* < 0.05. In each iteration, shadow features were randomly generated, and permutation tests were conducted to determine whether the importance of the original variables significantly exceeded that of the shadow features. The ‘confirmed’ variables were identified for each subgroup, and the differences between the two age groups were compared. Boruta analysis was performed in R using the Boruta package, with random forest as the base learner (ntree = 500, mtry = 3).

### 2.8. Statistical Analysis

All statistical analyses and modeling were conducted in R (v4.2.1). The following packages were used: randomForest (4.7–1.2) (random forest), e1071 (1.7–17) (support vector machine), pls (2.9) (partial least squares regression), Boruta (10.0.0) (feature selection), caret (7.0–1) (model training and cross-validation), FactoMineR (2.12) and factoextra (2.0.0) (principal component analysis), and ggplot2 (4.0.1) (graphical visualization). The significance level for all statistical tests was set at α = 0.05 (two-tailed). To determine whether differences in predictive accuracy among the PLSR, SVR, and RF model architectures were statistically significant, a two-tailed Diebold–Mariano (DM) test was performed on the paired sequences of squared prediction residuals, using the forecast package.

## 3. Results

### 3.1. Sample Characteristics

Descriptive statistics for the eight biochemical indicators and sensory scores of the 62 tomato germplasm accessions are presented in Table 1, while the full individual raw datasets for each cultivar have been compiled in Supplementary Table S2. Across the entire sample set, all measured indicators exhibited substantial variation ranges, indicating that the selected germplasm collection harbors considerable genetic diversity and is well suited for constructing cross-cultivar quality prediction models. Soluble solids content ranged from 3.5% to 11.2%; soluble sugar content ranged from 31.1 to 99.8 mg/g FW. Fructose and glucose contents ranged from 12.1 to 52.5 mg/g FW and from 4.0 to 23.0 mg/g FW, respectively, with marked differences in sugar composition ratios among accessions. Total acidity ranged from 0.094% to 1.38%; vitamin C content ranged from 2.7 to 13.8 mg/g FW. Total free amino acids and glutamic acid contents also showed substantial inter-accession variation, ranging from 1.95 to 4.43 μmol/g FW and from 0.47 to 1.12 mg/g FW, respectively. Sensory scores (all-panel mean) ranged from 2.10 to 4.23, spanning grades from ‘poor flavor’ to ‘excellent flavor,’ further confirming the representativeness of the material selection. These broad variation ranges provided sufficient input gradients for the subsequent model construction.

### 3.2. Sensory Score Distribution and Panel Reliability

**Figure 1:**
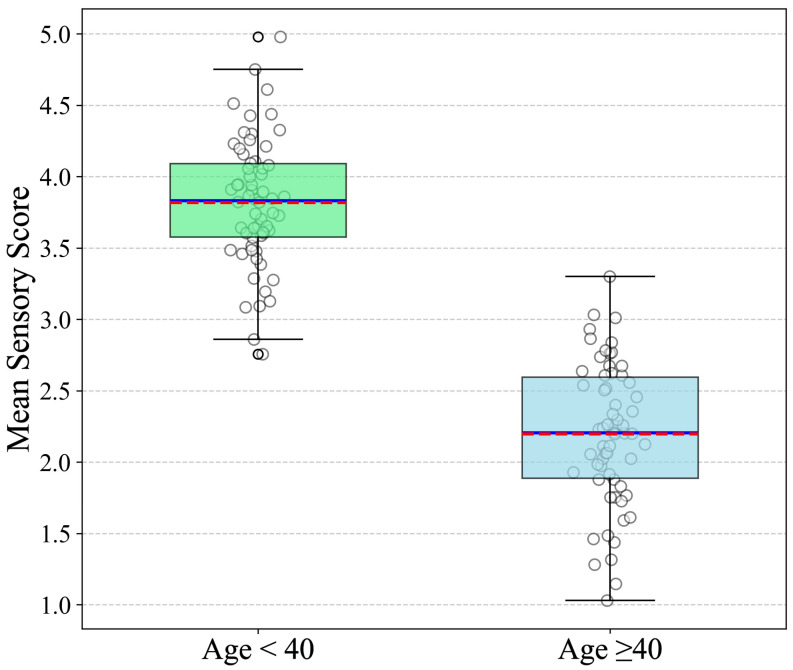
Box-and-whisker scatter plot of mean scores for the <40 years group and the ≥40 years group.

The distribution of mean sensory ratings across the 30 panelists and 62 samples approximated normality, with an overall mean of 3.12 ± 1.04. Upon age stratification, the <40 years group and the ≥40 years group exhibited markedly different mean score distributions (Figure 1): the <40 years group mean was 3.87 ± 0.15, and the ≥40 years group mean was 2.28 ± 0.21, with Welch’s *t*-test confirming the difference was highly significant (t = 47.46, *p* < 0.01). Retrospective power analysis revealed an effect size of Cohen’s d = 8.51 (*p* < 0.01), which minimizes the risk of Type II errors and supports the statistical robustness of the age-stratified machine learning inputs. This significant absolute mean shift (*p* < 0.01) indicates a strong systematic divergence in scale calibration and baseline quality expectations between the two generations, which is explored further in the Discussion Section. Kendall’s coefficient of concordance (W) analysis yielded the following results: within the <40 years group, W = 0.72 (*p* < 0.01, 95% CI: [0.65, 0.78]); within the ≥40 years group, W = 0.69 (*p* < 0.01, 95% CI: [0.61, 0.75]), indicating that both age subgroups achieved high internal agreement. The overall panel W = 0.56 (*p* < 0.01, 95% CI: [0.48, 0.63]), while lower than the within-group values, remained within the moderately acceptable range, suggesting the presence of a certain degree of systematic differential scoring between age groups, although the overall ranking trend was still broadly aligned. Taken together, these results demonstrate that the sensory evaluation data possess high reliability and stability, and that age stratification represents a potentially influential factor affecting flavor preference, warranting further investigation through modeling approaches.

### 3.3. Principal Component Analysis of Biochemical Indicators

**Figure 2:**
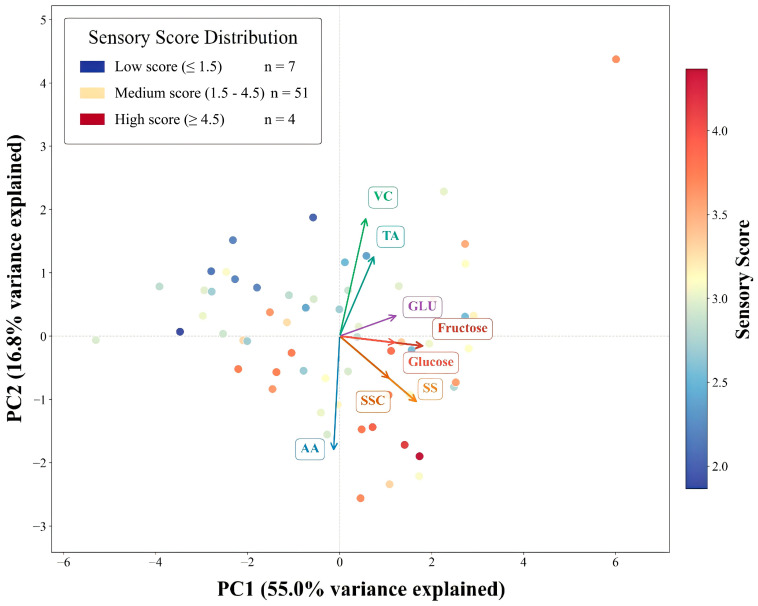
PCA biplot of biochemical indices and sensory scores for 62 tomato samples.

Principal component analysis (PCA) was conducted to explore the overall structural patterns of the eight biochemical indicators across the 62 tomato accessions. The first two principal components (PC1 and PC2) explained 55.0% and 16.8% of the total variance, respectively, with a cumulative contribution of 71.8%, collectively capturing the majority of the variance in the original variables. The biplot (Figure 2) revealed that fructose, glucose, and soluble sugar clustered tightly at the positive end of PC1, indicating strong correlations among these three indicators and a shared basis for sweetness-related metabolites. Total acidity was independently distributed at the negative end of PC2, forming an orthogonal relationship with the sweetness-related indicators. Soluble solids content was positioned between the positive end of PC1 and the negative end of PC2, reflecting its composite contribution from both sugars and acids. Total free amino acids and glutamic acid formed a small cluster at the negative end of PC1, suggesting that their variation patterns are relatively independent from those of the sugar and acidity indicators.

Upon overlaying the sensory scores as color codes onto the biplot, high-scoring samples (≥4.5) were found predominantly in regions associated with elevated fructose, glucose, and soluble sugar levels, whereas low-scoring samples (≤1.5) were distributed toward areas with higher total acidity or lower sugar indicators. These observations further demonstrate that tomato flavor quality is jointly modulated by multiple biochemical dimensions, and that a single indicator is insufficient to fully account for sensory scores, thereby providing a structural rationale for the subsequent multi-indicator modeling approach.

### 3.4. Model Performance—Overall Model

Using eight biochemical indices to predict sensory scores (overall mean), the predictive performances of three models, partial least squares regression (PLSR), support vector regression (SVR), and random forest (RF), were evaluated by leave-one-out cross-validation (LOOCV) and repeated 5-fold CV. The results are presented in Table 2. Random forest (RF) achieved the best performance across all evaluation metrics, with an R^2^ of 0.82, an RMSE of 0.29, and an MAE of 0.23 (CV R^2^ = 0.81 ± 0.05), indicating that this model can effectively capture the complex relationship between biochemical indices and sensory scores. PLSR showed intermediate performance (R^2^ = 0.71, RMSE = 0.38, MAE = 0.31, CV R^2^ = 0.67 ± 0.07), suggesting that while the linear model explains most of the variance, its predictive accuracy is lower than that of RF. SVR performed relatively poorly (R^2^ = 0.58, RMSE = 0.46, MAE = 0.37, CV R^2^ = 0.54 ± 0.10). Hyperparameter optimization via grid search selected the nonlinear Radial Basis Function (RBF) kernel as superior to the linear configuration, with final optimal hyperparameter coordinates settled at a cost penalty C = 10 and kernel width parameter γ = 0.12. Although the RBF kernel can capture some nonlinear relationships, its stability is compromised under small-sample conditions due to sensitivity to parameter selection. Furthermore, the out-of-bag (OOB) error of RF was 0.31, which is close to the cross-validated RMSE, indicating reasonable generalization performance. The two-tailed DM test validated that the ensemble RF model significantly outperformed the linear PLSR (DM statistic = 2.75, *p* < 0.05) and the kernel-based SVR model (DM statistic = 3.84, *p* < 0.05). This confirms that the RF architecture provides a statistically superior mapping function for multi-parameter biochemical-to-sensory translations compared to classical linear and kernel approaches.

The superior performance of the RF model compared to the other two highlights its enhanced capability to capture complex multi-variable interactions and non-additive patterns within the chemical–sensory matrix that traditional linear frameworks fail to adequately resolve. However, the residual unexplained variance (1 − R^2^ = 0.18) in the optimal RF model points to a structural limitation of the current indicator matrix: the absence of volatile organic compound (VOC) profiling. Because tomato flavor is an integrated sensory experience, this remaining error fraction likely represents the uncaptured aromatic components that heavily influence overall panelist perception.

### 3.5. Model Performance—Stratification by Age

To explore the effect of age on quality prediction, sensory scores of the <40 years group and the ≥40 years group were respectively used as target variables, and PLSR, SVR, and RF models were constructed following the same modeling procedure. The results are shown in Table 3. RF consistently achieved the best predictive performance in both age groups. For the <40 years group, the RF model yielded an R^2^ of 0.79, an RMSE of 0.32, and an MAE of 0.25 (CV R^2^ = 0.75 ± 0.09); for the ≥40 years group, the RF model achieved an R^2^ of 0.85, an RMSE of 0.26, and an MAE of 0.20 (CV R^2^ = 0.82 ± 0.08). Notably, the R^2^ for the ≥40 years group was substantially higher than that of the <40 years group, with correspondingly lower RMSE and MAE values, indicating that the biochemical indices predicted sensory scores more accurately for the ≥40 years group. PLSR and SVR metrics suggested that all modeling paradigms follow a uniform trend. ANOVA on MAEs of the two age groups and the three models confirmed that prediction errors in the ≥40 years group were significantly lower than in the <40 years group (F = 5.04, *p* < 0.05), and RF maintained a significant performance advantage over both PLSR and SVR across both age segments (F = 7.29, *p* < 0.05).

These findings suggest that age not only influences the absolute values of sensory scores but also modulates the mapping relationship between biochemical indices and sensory scores. The sensory scores of the ≥40 years group are more readily explained by biochemical indices, whereas the sensory scores of the <40 years group may involve more sources of variation not captured by the biochemical indices (e.g., differences in sensitivity to volatile aromatic compounds) or may reflect greater dispersion in taste preferences among individuals in the younger group.

### 3.6. Boruta-Based Variable Importance and Feature Correlation

**Figure 3:**
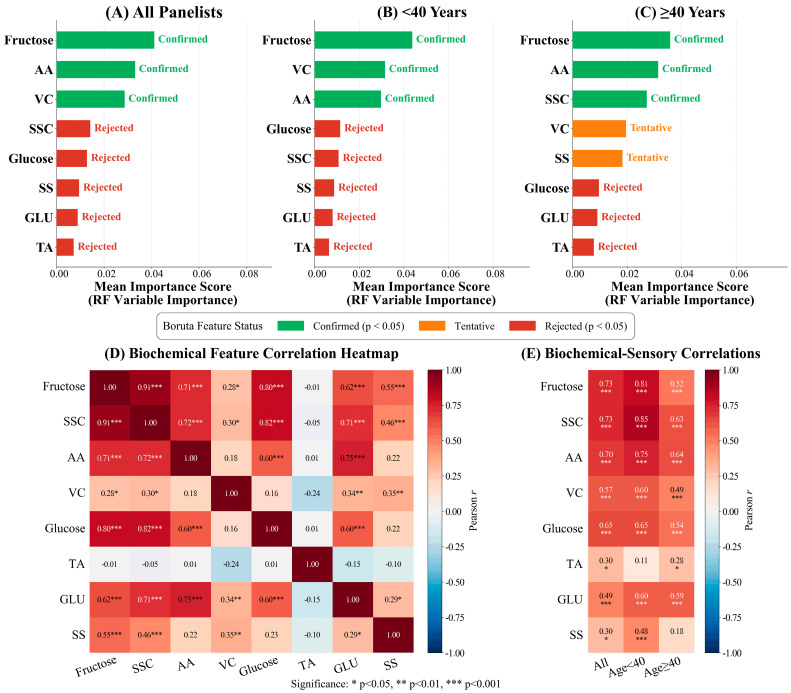
Boruta-based feature selection and feature correlation for key biochemical indicators in tomato sensory evaluation across different age groups. Specifically, (**A**) Boruta-confirmed variables and their importance rankings in the full panel model. (**B**) Boruta-confirmed variables and their importance rankings in the under-40 age group model. (**C**) Boruta-confirmed variables and their importance rankings in the 40-and-above age group model. (**D**) Biochemical feature correlation heatmap. (**E**) Biochemical–sensory correlation across different age groups.

To identify the key biochemical indicators driving sensory scores and to compare their differences across age groups, feature selection was performed using the Boruta algorithm. The Boruta algorithm was run based on a random forest model with the overall mean, the <40 years group scores, and the ≥40 years group scores as target variables, respectively. Feature-wide correlation and biochemical–sensory correlation across age groups were also conducted. The results are presented in Figure 3.

In the full-panel model, the Boruta algorithm identified fructose, total free amino acids, and vitamin C as important variables, all of which were designated as “Confirmed” (*p* < 0.05), indicating that these three indicators made significant contributions independent of shadow variables within the random forest model. In contrast, glucose, soluble sugars, soluble solids, glutamate, and total acidity were classified as “Rejected”, as they failed to reach the significance threshold.

After age stratification, clear differences in variable importance were observed. In the under-40 age group model, the confirmed variables were fructose, vitamin C, and total free amino acids (three variables designated as “Confirmed”), which were highly consistent with the full-panel results, with the importance ranking being fructose (0.0438) > vitamin C (0.0316) > total free amino acids (0.0298). In the age group of 40 years and above, the set of confirmed variables was adjusted to fructose, total free amino acids, and soluble solids (three “Confirmed” variables), whereas vitamin C and soluble sugars were classified as “Tentative”; the importance ranking was fructose (0.0358) > total free amino acids (0.0314) > soluble solids (0.0272).

Notably, sugars (fructose, glucose, SSC) and AAs demonstrated the strongest positive associations with sensory scores across different age groups (both age groups *r* = 0.52–0.85, *p* < 0.001), whereas SS and TA showed weak and mostly non-significant correlations, suggesting that sugar content and amino acids are the primary biochemical drivers of sensory perception, with some age-related variation in the relative importance of specific compounds. However, among the eight biochemical indicators, glutamate, glucose, and total acidity did not reach the confirmation threshold in any of the three models, while soluble sugars exhibited a marginal status in the ≥40 years group, indicating that these indicators contributed only limited independent effects to sensory scores after controlling for other variables. Total free amino acids, as the backbone compounds of umami taste, were confirmed as important drivers across all three models (All Panelists: 0.0331; <40 years: 0.0298; ≥40 years: 0.0314), demonstrating high robustness across age strata and further substantiating the substantial contribution of umami substances to tomato flavor quality. Fructose, a typical representative of sweet-tasting compounds, was also consistently confirmed in all three models with the highest importance scores (All Panelists: 0.0412; <40 years: 0.0438; ≥40 years: 0.0358), reaffirming the dominant role of sweet substances. In contrast, total acidity consistently ranked at the bottom across all models, suggesting that acid balance does not act as an independent dominant factor; rather, its role in flavor may involve a synergistic coupling with sweet substances to collectively modulate tomato flavor quality.

### 3.7. Relationship Between Key Driving Factors and Sensory Scores

**Figure 4:**
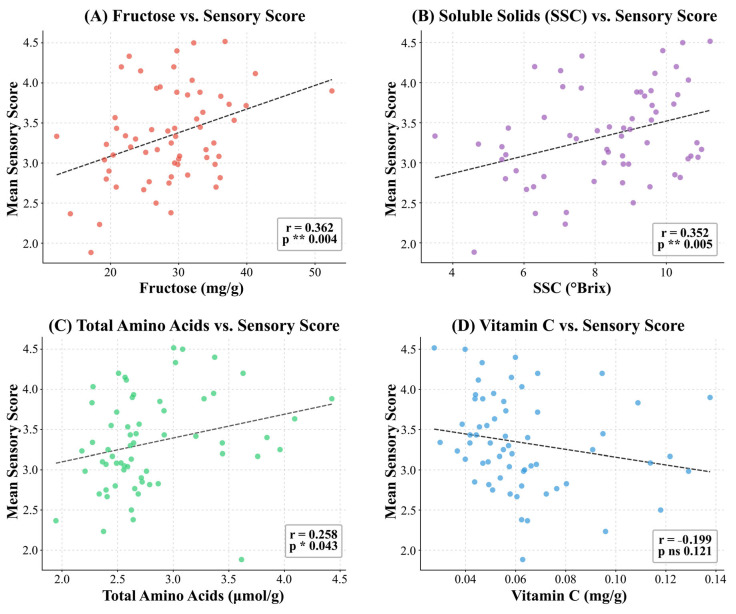
Relationship between key biochemical indicators and sensory scores. Specifically, (**A**) shows the relationship between fructose content and sensory scores; (**B**) shows the relationship between soluble solids content (SSC) and sensory scores; (**C**) shows the relationship between total free amino acids and sensory scores; and (**D**) shows the relationship between vitamin C content and sensory scores. **, * and ns represent significance levels *p* < 0.01, *p* < 0.05 and not significant, respectively.

To intuitively elucidate the relationships between the core driving factors and sensory scores, and to account for the superior performance of the random forest (RF) model over partial least squares regression (PLSR), scatter plots of fructose and soluble solids content against the overall mean sensory scores were constructed (Figure 4A,B), complemented by the analytical results for total free amino acids and vitamin C (Figure 4C,D).

Figure 4A illustrates the relationship between fructose content and sensory scores. The results indicated that fructose content increased alongside higher sensory scores, exhibiting distinct distribution patterns across different quality intervals. In the low-score range (2.0–3.0), fluctuations in fructose content corresponded to minimal score variance, manifesting as a vertical distribution pattern of data points within this interval. In the medium-to-high score range (3.0–4.0), fructose content showed a positive correlation with sensory scores (*r* = +0.362, *p* < 0.01). Notably, some samples with moderate fructose content but high soluble solids content achieved relatively high scores. This trend suggests that tomato flavor perception may depend on a co-dependent, multi-variable combination of indicators rather than the standalone, additive accumulation of single sugar components.

Figure 4B depicts the relationship between soluble solids content (SSC) and sensory scores. The data revealed a significant positive correlation between SSC and sensory scores (*r* = +0.352, *p* < 0.01), with a correlation pattern highly similar to that of fructose. This result is physiologically plausible: soluble solids in tomato fruit mainly consist of soluble sugars (fructose, glucose), organic acids, and some soluble proteins, among which sugars account for approximately 50% to 70%; therefore, SSC, as a comprehensive quality indicator, exhibits a contribution pattern statistically analogous to that of fructose.

Figure 4C presents the relationship between total free amino acids and sensory scores. The results revealed a weak positive correlation between total free amino acids and sensory scores (*r* = +0.258, *p* < 0.05). Notably, the samples with high free amino acid content (>3.0 μmol/g) exhibited a relatively concentrated trend of high scores (3.0–4.5) in the scatter plot, whereas the samples with low free amino acid content (<2.5 μmol/g) showed a wider distribution of scores (2.0–4.0). This segmented pattern suggests that the contribution of free amino acids to sensory quality may involve a threshold effect, i.e., their contribution to umami and flavor complexity becomes pronounced only when the amino acid content exceeds a certain critical level.

Figure 4D illustrates the relationship between vitamin C content and sensory scores. In contrast to sweet-tasting substances, vitamin C exhibited a weak negative correlation with sensory scores (*r* = −0.199, *p* > 0.05); although it was identified as a significant variable by the Boruta algorithm in the full-panel model, the scatter plot showed considerable dispersion. Further analysis revealed that the samples with high vitamin C content (>0.08 mg/g) displayed a bipolar distribution in sensory scores: some high-vitamin-C samples received relatively high scores (>3.5), whereas others scored low (<2.5). This phenomenon suggests that vitamin C does not act as a direct, independent driver of taste perception in the oral cavity. Because ascorbic acid functions primarily as a minor nutritional component rather than a dominant tastant like fructose or citric acid, high vitamin C concentrations can co-occur across vastly different baseline sugar and acid profiles. This accounts for the observed score dispersion and demonstrates that while the Boruta algorithm identifies vitamin C as a significant diagnostic marker for overall fruit quality, its predictive contribution is indirect rather than causative.

The aforementioned segmented and interactive nonlinear structure explains why linear models (e.g., PLSR) could not adequately fit this dataset. In this study, the relationship between fructose and sensory scores exhibited a clear interval-dependent pattern: the correlation was weak in the low-score range but became significantly stronger in the high-score range. Similarly, vitamin C and free amino acids displayed threshold effects or bipolar distribution characteristics. These complex nonlinear interaction patterns make it difficult for PLSR, which is based on global linear assumptions, to accurately capture the differential driving mechanisms across different quality levels. In contrast, random forest, through the ensemble of multiple decision trees, can adaptively identify local optimal split points in different regions of the feature space, thereby effectively capturing these segmented nonlinear relationships. This is also the fundamental reason why the Boruta algorithm can robustly identify feature importance within the random forest framework.

## 4. Discussion

Using 62 tomato germplasm resources as plant materials, this study systematically compared the performance of three models, partial least squares regression (PLSR), support vector regression (SVR), and random forest (RF), in predicting sensory scores based on biochemical indicators. Age stratification was introduced into tomato quality modeling, and the Boruta algorithm was integrated to identify key quality-driving factors. The findings indicate the nonlinear mapping relationships between biochemical indicators and sensory quality and offer perspectives and tools for the targeted flavor improvement of tomatoes tailored to different age-stratified groups. The following discussion is organized around five core themes.

### 4.1. Superior Performance of Random Forest and Its Biological Implications

Among the three models, random forest (RF) achieved the best performance across all evaluation metrics (R^2^ = 0.82, RMSE = 0.29, MAE = 0.23), whereas partial least squares regression (PLSR) (R^2^ = 0.71) and support vector regression (SVR) (R^2^ = 0.58) were both inferior to RF. The utility of the Boruta algorithm in this context serves as a robust tool to isolate independent metabolic contributions within a highly co-linear dataset, thereby revealing structurally distinct preference profiles between the age groups. The relatively poor performance of SVR in this study may be attributed to the small sample size (N = 62) and the difficulty of hyperparameter optimization: although the radial basis function (RBF) kernel is theoretically capable of handling nonlinear relationships, under conditions of a small sample size and a moderate-dimensional feature space (eight indicators), the grid search struggles to stably converge to the optimal parameter combination, leading to limited generalization ability [19]. As a linear method, PLSR achieved an R^2^ of 0.71, indicating that there is indeed a strong linear component between the biochemical indicators and sensory scores; nevertheless, approximately 29% of the variance remained unexplained by the linear model, confirming the presence of nonlinear structures in the data. Research [15,20] achieved an R^2^ of 0.71–0.79 using RF architecture for tomato and strawberry sensory prediction, noting that nonlinear ensembles consistently outperform traditional linear modeling when mapping complex chemical inputs.

The superior performance of random forest (RF), from a biological perspective, is not solely attributable to the inherent robustness of the algorithm; rather, it profoundly reflects the nonlinear and interactive mapping between biochemical indicators and sensory scores [20]. However, the relatively high predictive capacity achieved by this optimal framework warrants a rigorous evaluation regarding the potential risk of algorithmic overfitting, a common vulnerability when machine learning is applied to relatively modest agricultural datasets (N = 62). To confirm that this high accuracy level represents authentic biological signaling rather than the mathematical memorization of sample noise, several architectural safeguards must be highlighted. First, the RF architecture inherently suppresses variance through bootstrap aggregation (bagging), which was mathematically tightened by restricting individual decision tree depths to prevent over-parameterization. Second, our reported performance relies on LOOCV, a strict iterative protocol that isolates testing instances from the training loop, thereby penalizing artificial inflation of accuracy. This cross-validated stability is further validated by its close convergence with the OOB error (OOB R^2^ = 0.79). While these internal regularizations establish robust mathematical reliability for the current genetic pool, testing this framework against multi-environment cultivated tomatoes remains an essential next step to guarantee its global portability across diverse breeding programs.

The PCA biplot showed that fructose and glucose were highly clustered on the positive side of PC1 (PC1 = 55.0%, PC2 = 16.8%, cumulative variance 71.8%), whereas total acidity was distributed independently along PC2, suggesting that sweetness and acidity mutually modulate rather than simply additively determine flavor quality. The scatter plots further confirmed that the responses of sensory scores to fructose (*r* = +0.362, *p* < 0.01) and soluble solids (*r* = +0.352, *p* < 0.01) were not simple monotonic linear relationships. In the medium-to-high score range (3.0–4.0), the positive correlation between sugar content and sensory scores was more pronounced, whereas in the low-score range (2.0–3.0), the marginal benefit of increasing sugar content to score improvement was rather limited. Total free amino acids also exhibited a threshold effect: samples with high free amino acid content (>3.0 μmol/g) showed a concentrated trend of high scores, whereas samples with low free amino acid content exhibited a wider distribution of scores. Vitamin C showed a weak negative correlation with sensory scores (*r* = −0.199) and displayed a bipolar distribution pattern in the scatter plot.

These complex nonlinear interaction patterns make it difficult for the PLSR model, which is based on global linear assumptions, to accurately capture the differentiated driving mechanisms across different quality levels. In contrast, random forest, through the ensemble of multiple decision trees, can adaptively identify local optimal split points in different regions of the feature space, thereby effectively capturing these segmented nonlinear relationships. Moreover, the out-of-bag (OOB) error of RF (0.31) was very close to the cross-validated RMSE (0.29), further validating the reliability of its generalization ability. Therefore, the modeling advantage of RF is consistent with the inherent biological complexity underlying tomato flavor quality formation, rather than merely a comparison of computational methods.

### 4.2. Breeding Significance of Fructose and Total Free Amino Acids as Core Drivers of Fruit Quality

The results of Boruta variable selection revealed an important finding: both fructose and total free amino acids were confirmed as important variables in all three model groups (three “Confirmed” designations), and their importance scores exhibited high stability across the three groups: the importance scores for fructose were 0.0412 (full panel), 0.0438 (<40 group), and 0.0358 (≥40 years group), while those for total free amino acids were 0.0331 (full panel), 0.0298 (under-40 group), and 0.0314 (≥40 years group). This cross-age consistency was observed for only these two out of the eight biochemical indicators examined in this study, indicating that they constitute robust core factors driving tomato flavor quality.

From a physiological perspective, fructose, as the soluble sugar with the highest sweetness and the greatest genotypic variation in ripe tomato fruit (content range in this study: 12.1–52.5 mg/g FW), exhibits a dominant role that is highly consistent with human taste physiology: fructose is approximately 1.7 times sweeter than sucrose, with rapid onset and offset of sweetness, delivering a clean and refreshing sweet sensation. Consequently, fructose emerges as a promising candidate for consideration as a primary selection target in flavor breeding. Total free amino acids, as a comprehensive indicator of umami backbone compounds, also display robust contributions across age strata with a well-grounded biological basis: amino acids in tomatoes not only serve as important sources of umami but also participate in the accumulation of precursors for the Maillard reaction, positively regulating overall flavor complexity. Furthermore, as a multi-component composite indicator, amino acids with positive and negative taste effects may synergize at the total content level, enabling the integrated effect to surpass the independent contributions of individual components.

Age-stratified analysis further revealed the differentiated contribution patterns of vitamin C and soluble solids. In the under-40 age group, vitamin C was confirmed as the third most important variable (importance score = 0.0316), suggesting a hypothesis that younger assessors may assign greater perceptual weight to nutritionally active compounds such as vitamin C, although direct measurements of sensitivity to such compounds were not conducted. In the group aged 40 years and above, soluble solids were independently confirmed as the third most important variable (0.0272), whereas vitamin C was downgraded to a tentative status in this subgroup. This reversal carries important biological implications: soluble solids, as a comprehensive reflection of sugars, acids, and other soluble substances in ripe tomato fruit (with sugars accounting for approximately 50% to 70%), provide more stable explanatory power for the flavor perception of older assessors; in contrast, younger assessors may be more sensitive to the perception of antioxidant compounds, thereby making vitamin C an independent contributing factor for this group.

The above findings may offer a preliminary reference for breeding practice. Specifically, fructose and total free amino acids, given their consistent confirmation across age-stratified models, warrant consideration as candidate screening indicators in flavor quality improvement programs. Furthermore, differentiated strategies can be implemented for specific market segments: for young assessors, the potential contribution of nutritionally active compounds such as vitamin C to sensory quality could be explored; for middle-aged and elderly assessors, the combined optimization of soluble solids and fructose may merit prioritization in trait selection. It is recommended that rapid detection techniques such as near-infrared (NIR) spectroscopy be introduced in early breeding generations to enable the simultaneous, non-destructive determination of fructose and total free amino acids, thereby improving the accuracy and efficiency of flavor-directed selection.

### 4.3. Interpretation of the Contribution Mechanisms of Other Biochemical Indicators

This study clarifies the age-stratified contribution mechanisms of total free amino acids and vitamin C to tomato flavor perception. Total free amino acids, the molecular backbone of umami, were confirmed by Boruta as stable drivers across all models (importance scores: full panel: 0.0331; <40: 0.0298; ≥40 years: 0.0314). This consistency indicates that their role in flavor complexity is robust across age groups. Distribution analyses suggest an amino acid threshold effect; when content exceeds approximately 3.0 μmol/g, high sensory scores become heavily concentrated. This reinforces the value of targeting elevated free amino acid levels in quality breeding to enhance taste depth without shifting baseline sweetness.

Conversely, the relationship between vitamin C and sensory is more complex [21]. While selected by Boruta in the full-panel model (importance = 0.0287), it correlated weakly with sensory scores (*r* = −0.199, *p* > 0.05) and exhibited a highly dispersed, bipolar distribution at concentrations exceeding 0.08 mg/g. This indicates that vitamin C is not a direct oral tastant; its presence can co-occur with either highly optimized or poorly balanced sugar–acid matrices. Notably, vitamin C was downgraded to a tentative status in the ≥40 years group. This demographic shift aligns with a study demonstrating that older adults experience a gradual decline in olfactory and gustatory acuity [22]. Consequently, older cohorts’ hedonic and quality evaluations are likely to depend more heavily on the primary, high-amplitude inputs of the core sugar–acid matrix to register flavor satisfaction, rendering ascorbic acid predictive only within younger cohorts [23].

Among the rejected indicators, glutamate was a single umami amino acid, whereas total free amino acids were confirmed. The relatively low feature importance assigned to glutamic acid (GLU) appears counterintuitive given its established role in tomato umami taste, but this reflects a clear distinction between biological utility and algorithmic selection. In tree-based ensembles like RF, individual sub-components are often downweighted when a closely related aggregate trait is evaluated simultaneously. In the indicator matrix, GLU represents a highly stable constituent within the broader total free amino acid pool (AA mean = 2.798 μmol/g FW, CV = 0.181). Furthermore, GLU exhibited a tightly constrained phenotypic distribution across all samples (ranging from 0.466 to 1.124 mg/g, CV = 0.228,). This limited variance indicates that baseline umami thresholds were uniformly satisfied across the tomato samples, minimizing the statistical leverage of GLU as a discriminatory variable during node splits. Consequently, while biologically essential for basic palatability, GLU functions as a metabolic prerequisite in this model rather than a primary statistical differentiator for ranking premium flavor tiers. When amino acids with positive and negative taste effects coexist, the independent contribution of a single component is attenuated, whereas the integrated effect at the total level may achieve statistical significance. Taken together, these results indicate that, within the scope of the eight indicators selected in this study, the sweet-tasting compound (fructose) and the umami backbone compounds (total free amino acids) are the core factors dominating flavor quality, whereas the contribution mechanism of vitamin C involves a complex trade-off between antioxidant protection and flavor perception. Future studies may further quantify volatile aroma compounds and incorporate them into models to more comprehensively decipher the multidimensional structure of tomato flavor quality.

A primary limitation of the current indicator matrix is the exclusion of volatile profiling. Volatile organic compounds, such as hexanal, 2-isobutylthiazole, and β-ionone, interact synergistically with soluble sugars and organic acids to enhance or alter perceived sweetness and aroma intensity. Because our input feature space lacks these olfactory drivers, the models function strictly as taste-matrix predictors rather than comprehensive flavor-profile simulators. Future iterations of this framework must integrate high-throughput volatile profiling (e.g., via GC-MS) to capture these multi-sensory interactions and resolve the remaining predictive variance. The unexplained variance in our under-40 prediction model (R^2^ = 0.79) likely reflects this omitted olfactory layer, suggesting that younger assessors may weigh volatile-taste interactions more heavily than older ones.

### 4.4. Differences in Age-Stratified Models and Their Implications for Market-Segmented Breeding

This study introduced age stratification into tomato quality modeling. While the age-stratified subgroup sizes (N = 14 and N = 16) would be limited for hedonic consumer testing, they provide high statistical robustness within a descriptive analysis framework. In accordance with ISO 8586 and Quantitative Descriptive Analysis (QDA) standards, a highly calibrated panel of 8 to 12 experts is sufficient to establish analytical reproducibility. By employing intensive alignment protocols to minimize individual variance, each age-stratified subgroup functions as an independent, statistically powered sensory instrument that exceeds standard panel size mandates. The prediction accuracy of the ≥40 years group model (R^2^ = 0.85, RMSE = 0.26, MAE = 0.20) was superior to that of the under-40 group model (R^2^ = 0.79, RMSE = 0.32, MAE = 0.25), and the same trend was observed for PLSR and SVR. The mean sensory scores differed significantly between the two groups (<40 group: 3.87 ± 0.15; ≥40 years group: 2.28 ± 0.21, t = 47.46, *p* < 0.01). The structural limitations of our sample size (N = 62) necessitate caution regarding global model generalization, suggesting that the high convergence between the LOOCV R^2^ (0.82) and the OOB error (0.31) only demonstrates internal stability and robustness within this germplasm pool. Kendall’s coefficient of concordance analysis indicated high consistency within each age group (<40 group: W = 0.72; ≥40 years group: W = 0.69), with the overall concordance (W = 0.56) falling within a moderate and acceptable range. This difference in itself suggests a systematic divergence in flavor preferences between different age groups, carrying both theoretical and practical significance.

The substantial divergence in absolute mean scores (3.87 vs. 2.28) points to a multi-faceted psychometric and experiential shift between generations that extends beyond progressive physiological receptor degradation. This magnitude indicates that the divergence most likely reflects a composite of differential scale-use behavior, wherein trained assessor groups adopt systematically different regions of the rating scale, and a smaller genuine perceptual component. One plausible explanation is that older assessors (≥40) possess an internalized quality standard calibrated against traditional or heirloom cultivars with rich, complex volatile matrices, anchoring their scale more stringently. Conversely, younger ones (<40 years), exposed primarily to modern supermarket varieties optimized for shelf-life, operate under a lower baseline benchmark. Furthermore, age-related declines in olfactory acuity have been reported to potentially alter cross-modal flavor integration [10]; such changes might contribute to the observed pattern whereby older assessors assign lower ratings when soluble taste indicators fail to exceed elevated perception thresholds.

While *z*-score normalization within each subgroup would eliminate the mean offset, it would also remove the absolute scoring information that carries practical relevance. Because within-group Kendall’s concordance values remained highly robust (<40 group: W = 0.72; ≥40 years group: W = 0.69), this gap represents a uniform psychometric scale-offset rather than panel inconsistency. This internal alignment explains why the random forest models achieved high predictive accuracies within each age-stratified group (R^2^ = 0.79 and 0.85, respectively), validating the mathematical robustness of the underlying biochemical-to-perceptual mapping.

From a physiological perspective, changes in taste sensitivity with age are a common phenomenon. Human aging is characterized by a gradual decline in taste sensitivity, particularly for sweetness and umami, which directly corresponds to a documented contraction in the density of functional fungiform papillae and a disruption in taste bud cell-renewal kinetics [24]. At the molecular level, this phenotypic shift is driven by the progressive downregulation of transcripts encoding the heterodimeric G-protein coupled receptors T1R2 and T1R3, which serve as the primary peripheral gatekeepers for sweet taste transduction. Furthermore, recent advancements in gustatory biology indicate that age-related structural degradation of taste buds is frequently exacerbated by elevated apoptosis and impaired homeostasis of supporting cells within the gustatory epithelium [25,26]. Consequently, it is plausible that such physiological changes contribute to the observation that the ≥40 group exhibits a significantly higher absolute taste detection threshold. The residual variance not explained by biochemical indicators in this group may be associated with volatile aroma compounds, textural attributes, or more sensitive differences in taste, leading to a relatively lower R^2^ for the model.

This finding may have preliminary implications for market-segmented breeding. If the breeding target is aimed at markets dominated by middle-aged and elderly populations (e.g., traditional wet markets, daily household consumption), the synergistic accumulation of fructose and total free amino acids could be prioritized in trait selection, with quality screening supported by basic biochemical indicator assays. If the target is younger groups (e.g., premium supermarkets, ready-to-eat tomatoes, salad tomatoes), a comprehensive balance of sweetness, vitamin C, and aroma compounds may be beneficial. Notably, the confirmed variables in the ≥40 years group model included soluble solids (0.0272), whereas vitamin C was assigned a tentative status; this feature can serve as a basis for differentiated screening targeting different demographic groups. Future breeding programs may consider exploring a ’customized’ quality evaluation and screening system based on the age structure of target market segments to meet market demands with greater precision.

### 4.5. Translational Considerations for Breeding Practice

Based on the results of the multi-model comparison and age-stratified analysis presented above, this study proposes the following specific recommendations for the targeted improvement of tomato flavor quality.

With regard to the breeding indicator system, this study found that fructose was confirmed in all three model groups with the highest importance scores (full panel: 0.0412; under-40: 0.0438; ≥40 years: 0.0358), indicating that it exerts a robust driving effect across age groups. Fructose content exhibited substantial variation among different genotypes (12.1–52.5 mg/g FW), suggesting high potential for genetic improvement. If these patterns are confirmed in multi-environment trials, rapid detection techniques such as near-infrared (NIR) spectroscopy could be considered for early breeding generations to enable an efficient, non-destructive determination of fructose content as part of a screening strategy.

Regarding the regulation of umami backbone compounds, total free amino acids were consistently identified as an important predictor by the Boruta algorithm in this study, indicating that their contribution to tomato flavor quality is robust across age strata. Samples with high free amino acid content (>3.0 μmol/g) exhibited a concentrated trend of high scores in the scatter plot, suggesting that focusing on increasing total free amino acids during variety selection may impart greater flavor complexity to the product. Furthermore, this study found that glutamate as a single component was rejected, whereas total free amino acids were confirmed, indicating that optimizing the proportion of amino acid composition may be more important than merely pursuing total content.

Regarding differentiated strategies for demographic groups, the comparison of age-stratified models revealed that the quality drivers were modulated differently between the two consumer groups. In the ≥40 years group, the confirmed variables were fructose, total free amino acids, and soluble solids, with vitamin C showing a tentative status; whereas in the under-40 group, the confirmed variables were fructose, vitamin C, and total free amino acids, with soluble solids not independently confirmed. Accordingly, for variety development targeting the middle-aged and elderly market segments, fructose, soluble solids, and total free amino acids may serve as core screening indicators. For the younger group, attention could be paid to the synergistic accumulation of nutritionally active components such as vitamin C, in order to cover the quality preferences of this group for health-oriented attributes.

With regard to the refinement of screening indicators, glucose and soluble sugars were excluded by Boruta in all three model groups of this study. This result suggests that although glucose exhibited a marginally significant positive correlation with sensory scores (*r* = +0.310, *p* < 0.05), its independent contribution can be substituted by fructose. Glucose and fructose are highly collinear in the sugar metabolic pathway, and the random forest model, under multivariate interactions, tends to retain fructose, which contributes more significantly to sweetness, while excluding glucose. Therefore, in practical breeding screening, directly determining fructose content alone may offer a more resource-efficient screening approach than measuring both fructose and glucose. Similarly, total acidity consistently ranked at the bottom across all models, suggesting that acid balance does not act as an independent dominant factor; rather, its role in flavor may involve synergistic coupling with sweet compounds. Future studies may explore whether the sugar-to-acid ratio provides greater explanatory power than total acidity alone in capturing the modulatory effect of acidity on flavor perception.

Although the machine learning workflows optimized in this study demonstrate a robust predictive capacity, several inherent limitations must be acknowledged. First, the feature space was strictly restricted to eight primary non-volatile biochemical components. While soluble sugars and organic acids dictate the baseline taste matrix of tomato fruit, complete flavor perception relies heavily on complex, multi-component volatile profiles that trigger retronasal olfaction. The absence of volatile compounds profiling represents an explicit limitation, as volatile–nonvolatile synergistic interactions could alter sensory scores. Second, the sample size constrained our ability to sequester a completely independent external validation cohort from a separate growing season or distinct climate zone. Consequently, while internal cross-validation and out-of-bag error configurations mathematically mitigated overfitting within this dataset, the geographic and temporal localization of the crop sample means that model portability to widely divergent environmental supply chains remains to be validated.

## 5. Conclusions

Based on the individual flavor-associated compounds reported previously, the principal contribution of this study lies in demonstrating a reproducible analytical framework that integrates nonlinear machine learning with age-stratified sensory evaluation to extract interpretable predictor importance rankings from a high-collinearity biochemical dataset. The findings conclude that fructose and total free amino acids consistently emerged as core predictors across all models and age stratification underscores their role as stable, high-value targets for flavor-oriented breeding programs. Meanwhile, the age-dependent divergence in biochemical indicator importance provides empirical support for incorporating age stratification into sensory modeling workflows. Crucially, the findings establish that assessor age functions as a structural determinant of taste perception within a trained descriptive panel framework, revealing that older cohorts display more uniform and predictable sensory responses tightly coupled to primary chemical inputs due to age-related gustatory shifts. Ultimately, these insights bridge the gap between fruit crop metabolomics and sensory-informed breeding strategy, providing an empirical foundation for market-segmented precision breeding programs. Future work should prioritize volatile compounds integration, multi-environment validation with independent test sets, and extension to direct consumer acceptance to assess whether the predictor importance patterns identified through trained panel evaluation translate to real-world purchasing and consumption behavior.

## Figures and Tables

**Table 1:** Descriptive statistics of biochemical and sensory indicators of tomato germplasm resources (N = 62).

Indicator	Unit	Mean	Standard Deviation	Coefficient of Variation	Minimum	Maximum
Fructose	mg/g FW	28.502	7.123	0.250	12.097	52.482
Glucose	mg/g FW	13.137	3.870	0.295	3.995	22.964
Soluble Sugar (SS)	mg/g FW	73.748	16.663	0.226	31.136	99.816
Vitamin C (VC)	mg/g FW	0.063	0.024	0.382	0.027	0.138
Soluble Solids Content (SSC)	°Brix	8.297	1.875	0.226	3.500	11.230
Total Free Amino Acids (AAs)	μmol/g FW	2.798	0.507	0.181	1.945	4.426
Glutamic Acid (GLU)	mg/g FW	0.760	0.174	0.228	0.466	1.124
Total Acidity (TA)	%	0.252	0.164	0.650	0.094	1.380
Sensory Score (<40 years subgroup mean)	-	3.867	0.583	0.151	2.303	4.909
Sensory Score (≥40 years subgroup mean)		2.284	0.486	0.213	1.324	3.294

**Table 2:** Comparison of predictive performance of the three models under LOOCV and repeated 5-fold cross-validation.

Models	LOOCV R^2^	LOOCV RMSE	LOOCV MAE	Repeated 5-Fold CV R^2^	Repeated 5-Fold CV RMSE	Repeated 5-Fold CV MAE
PLSR	0.71	0.38	0.31	0.67 ± 0.07	0.40 ± 0.09	0.28 ± 0.11
SVR	0.58	0.46	0.37	0.54 ± 0.10	0.49 ± 0.13	0.39 ± 0.09
RF	0.82	0.29	0.23	0.81 ± 0.05	0.31 ± 0.06	0.27 ± 0.04

**Table 3:** Predictive performance of the three models for different age groups.

Age Groups	Models	LOOCV R^2^	LOOCV RMSE	LOOCV MAE	Repeated 5-Fold CV R^2^	Repeated 5-Fold CV RMSE	Repeated 5-Fold CV MAE
<40 years old	PLSR	0.66	0.40	0.37	0.65 ± 0.09	0.40 ± 0.09	0.38 ± 0.08
	SVR	0.56	0.49	0.41	0.54 ± 0.11	0.50 ± 0.11	0.42 ± 0.10
	RF	0.79	0.32	0.25	0.75 ± 0.09	0.36 ± 0.11	0.27 ± 0.06
≥40 years old	PLSR	0.73	0.35	0.28	0.70 ± 0.08	0.39 ± 0.08	0.29 ± 0.07
	SVR	0.64	0.37	0.30	0.62 ± 0.10	0.42 ± 0.10	0.33 ± 0.08
	RF	0.85	0.26	0.20	0.82 ± 0.08	0.29 ± 0.07	0.21 ± 0.03

## Data Availability

The original contributions presented in this study are included in the article/Supplementary Materials. Further inquiries can be directed to the corresponding authors.

## Supplementary Materials

The following supporting information can be downloaded at https://www.mdpi.com/article/10.3390/foods15132358/s1, Table S1: Information about tomato samples; Table S2: Biochemical indicators and sensory scores of all samples.

## Abbreviations

The following abbreviations are used in this manuscript:

AAs	Total free amino acids
ANOVA	Analysis of variance
CIs	Confidence intervals
DM test	Diebold-Mariano test
FW	Fresh weight
GC-MS	Gas chromatography-mass spectrometry
GLU	Glutamic acid
LOOCV	Leave-one-out cross-validation
MAE	Mean absolute error
OOB	Out-of-bag
PCA	Principal component analysis
PLSR	Partial least squares regression
R^2^	Coefficient of determination
QDA	Quantitative descriptive analysis
RF	Random forest
RMSE	Root mean squared error
SS	Soluble sugar
SSC	Soluble solids content
SVR	Support vector regression
TA	Total acidity
VC	Vitamin C
W	Kendall’s coefficient of concordance
